# Whole-Genome Sequencing Analysis of Nontyphoidal *Salmonella enterica* of Chicken Meat and Human Origin Under Surveillance in Sri Lanka

**DOI:** 10.1089/fpd.2018.2604

**Published:** 2019-07-09

**Authors:** Moon Y.F. Tay, Sujatha Pathirage, Lakshmi Chandrasekaran, Uddami Wickramasuriya, Nirasha Sadeepanie, Kaushalya D.K. Waidyarathna, Liyanaralalage Dilini Chathurika Liyanage, Kelyn L.G. Seow, Rene S. Hendriksen, Masami T. Takeuchi, Joergen Schlundt

**Affiliations:** ^1^ School of Chemical and Biomedical Engineering, Nanyang Technological University (NTU), Singapore, Singapore.; ^2^ Nanyang Technological University Food Technology Centre (NAFTEC), Nanyang Technological University (NTU), Singapore, Singapore.; ^3^ Enteric Reference Laboratory, Department of Bacteriology, Medical Research Institute (MRI), Colombo, Sri Lanka.; ^4^ National Food Institute, Technical University of Denmark, Kongens Lyngby, 2800, Denmark.; ^5^ Food and Agriculture Organization of the United Nations (FAO), Rome, Italy.

**Keywords:** Salmonella enterica, CTX-M-15, whole genome sequencing, Sri Lanka, human, chicken meat, surveillance

## Abstract

A total of 73 nontyphoidal *Salmonella enterica* isolates, 33 from raw chicken meat and 40 from routine clinical specimens, were collected between 2015 and 2017 from eight cities in Sri Lanka for a pilot study of whole-genome sequencing for *Salmonella* surveillance. The isolates were characterized by conventional serotyping and whole-genome sequencing. The raw sequenced data were assembled and analyzed to predict *Salmonella* serotypes, determine sequence type (ST) profiles of genome and plasmid, and identify plasmid replicon sequences and antimicrobial resistance (AMR) genes. The most common serovar isolated from chicken meat was *Salmonella enterica* serovar Agona of ST13 (*n* = 16), in contrast to *Salmonella enterica* serovar Enteritidis of ST11 (*n* = 21) in human. *Salmonella enterica* serovar Corvallis is the only serovar that was overlapping between human and chicken meat. The level of agreement between serotyping and serotype prediction results was 100%. Among the 33 chicken isolates, multidrug resistance (MDR) was observed in five isolates, including two *Salmonella enterica* serovar Kentucky ST314, which harbored six different classes of AMR determinants. Among the 40 human isolates, MDR was detected in two *Salmonella enterica* serovar Chester (ST2063) isolates containing five different antibiotic classes of AMR determinants. Out of 73 isolates, the only human *Salmonella enterica* serovar Typhimurium strain of ST36 was found to possess extended-spectrum beta-lactamase (ESBL) gene, bla_CTX-M-15_, and it was positive for ESBL production. In summary, this study identified *S*. *enterica* serovars that were dominating in chicken meat and human and showed the genomic differences among the chicken meat and human strains. It should be noted that the limited number of isolates and sampling at a different time period means that thorough source attribution is not possible. To the best of our knowledge, this is the first report on the use of whole-genome sequencing analysis of nontyphoidal *S*. *enterica* isolated from chicken meat and human in Sri Lanka.

## Short Report/Case Study

*S**almonella enterica*, a common foodborne pathogen worldwide, has >2600 serovars that can cause infections of varying severity to human and animal. The nontyphoidal *Salmonella* (NTS) strains may be host generalist with broad host specificity that colonizes or infect a wide range of vertebrate animals or may be restricted to particular animal species (Feasey *et al.*, 2012). NTS infections usually cause mild to moderate self-limiting gastroenteritis in young adults, and no antibiotic treatment is required. However, in ∼6% of the gastroenteritis cases, bacteria may proceed to cause an invasive extraintestinal disease leading to bacteremia and focal infection in the young, elderly, and immunocompromised humans, and ciprofloxacin and extended-spectrum cephalosporin are commonly prescribed to treat such invasive disease (Rowe *et al.*, 1997). Globally, an increasing prevalence of ciprofloxacin and extended-spectrum cephalosporin resistance have been reported in clinical NTS strains (Crump *et al.*, 2015), and it is thought to be associated with the use of fluoroquinolones and beta-lactams as a growth promoter in food-producing animals. NTS is transmitted through animal products (mainly through eggs, meats, and poultry products) and produce contaminated with animal feces and/or human sewage, and contact with animals and animal environment (Crump *et al.*, 2015).

Ministry of Health in Sri Lanka reported an overall decreasing trend in the incidence of dysentery, enteric fever, and food poisoning for the period of 2007–2017 (MoH, 2018). The number of aforementioned foodborne illness cases that can be attributed to *S. enterica* in Sri Lanka is unknown. Similarly, the transmission pattern of *S. enterica* in Sri Lanka remains unclear, and there is no published data characterizing the molecular epidemiology of *S. enterica* in human and poultry production. In Sri Lanka, both fluoroquinolones and beta-lactams are used to treat human *Salmonella* infection and are banned for growth promotion purposes (personal communication from Dr. Palika Fernando, National AMR steering committee member, Head Department of Bacteriology, Veterinary Research Institute, Sri Lanka). Given the public health significance of *Salmonella*, this pilot cross-sectional genomic-based surveillance study is done to provide the NTS situation in humans and raw chicken meats from eight cities in Sri Lanka. It is important to note that this study is not designed to compare the prevalence between the different cities. On the contrary, this study aims to provide a molecular snapshot of genetic variability among the collected *Salmonella* strains.

A total of 73 nontyphoidal *S. enterica* isolates, 33 from raw chicken meat and 40 from clinical specimens (i.e., stool, blood, and joint fluid) were collected from eight cities in Sri Lanka, namely Awissawella, Badulla, Colombo, Galle, Jaffna, Kandy, Peradeniya, and Ragama between 2015 and 2017 (Table 1). Genomic DNA extraction, library construction, and sequencing were performed as previously described (Guo *et al.*, 2019). Sequence data were deposited into GenBank under BioProject accession number PRJNA504925. GenBank accession numbers for individual isolates are listed in Table 1. *De novo* assembly of draft genome (Afgan *et al.*, 2018), assessment of draft genome assembly quality (Gurevich *et al.*, 2013), and genomic analyses (Larsen *et al.*, 2012; Zankari *et al.*, 2012; Carattoli *et al.*, 2014; Zhang *et al.*, 2015; Alikhan *et al.*, 2018) were performed as previously described (Tay *et al.*, 2019). Conventional serotyping according to Kauffman–White scheme was done in-house at the Enteric Reference Laboratory in Medical Research Institute with antisera purchased from S&A Reagents Lab Ltd., Part. (Thailand).

**Table 1.** Whole-Genome Sequencing Characterization of 73 Nontyphoidal *Salmonella enterica* That Were Isolated from the Raw Chicken and Human in Various Cities in Sri Lanka

	*Laboratory identifier*	*Isolate*	*Sample type*	*Location*	*Sample isolation date*	*MLST*^a^	*Serotyping*^b^	*Predicted serotype(s)*^c^	*Resistance genes*^d^	*Point mutation relating to resistance*^d^		*Plasmid replicons*^e^	*pMLST*^f^		*GenBank accession*	*No. of Contigs (≥500 bp)*^g^	*Total length (≥500 bp)*^g^
										*gyrA*	*gyrB*		*IncF*	*IncI1*			
1	NAFTEC00025	SL_1_03	Raw chicken	Colombo	January 5, 2015	13	Agona	Agona	*fosA7*						SMON00000000	34	4838342
2	NAFTEC00026	SL_2_05	Raw chicken	Colombo	January 5, 2015	13	Agona	Agona	*fosA7*						SMOO00000000	33	4837977
3	NAFTEC00027	SL_3_07	Raw chicken	Colombo	January 5, 2015	13	Agona	Agona	*fosA7*			IncI1		ST-284	SMOP00000000	34	4928605
4	NAFTEC00028	SL_4_08	Raw chicken	Colombo	January 5, 2015	13	Agona	Agona	*fosA7*			IncI1		ST-284	SMOQ00000000	35	4928795
5	NAFTEC00029	SL_5_09	Raw chicken	Colombo	January 5, 2015	13	Agona	Agona	*fosA7*			IncI1		ST-284	SMOR00000000	39	4946197
6	NAFTEC00030	SL_6_10	Raw chicken	Colombo	January 12, 2015	13	Agona	Agona	*fosA7*			IncI1		ST-284	SMOS00000000	35	4929171
7	NAFTEC00031	SL_7_12	Raw chicken	Colombo	January 12, 2015	13	Agona	Agona	*fosA7*			IncI1		ST-284	SMOT00000000	31	4927977
8	NAFTEC00032	SL_8_13	Raw chicken	Colombo	January 12, 2015	13	Agona	Agona	*fosA7*			IncI1		ST-284	SMOU00000000	33	4928734
9	NAFTEC00033	SL_9_15	Raw chicken	Colombo	January 12, 2015	13	Agona	Agona	*fosA7*			IncI1		ST-284	SMOV00000000	32	4928272
10	NAFTEC00034	SL_10_20	Raw chicken	Colombo	January 12, 2015	13	Agona	Agona	*fosA7*			IncI1		ST-284	SMOW00000000	33	4945282
11	NAFTEC00035	SL_11_62	Raw chicken	Colombo	January 12, 2015	314	Kentucky	Kentucky	*aph(6)-Id, tet(A), blaTEM-1B, qnrS1, sul3*			IncX1			SMOX00000000	30	4704948
12	NAFTEC00036	SL_12_81	Raw chicken	Colombo	February 16, 2015	1541	Corvalis	Corvallis or Chailey	*qnrS1*						SMOY00000000	34	4894025
13	NAFTEC00037	SL_13_91	Raw chicken	Colombo	February 16, 2015	1541	Corvalis	Corvallis or Chailey	*qnrS1*			IncI1			SMOZ00000000	35	4984442
14	NAFTEC00038	SL_14_93	Raw chicken	Colombo	February 16, 2015	1541	Corvalis	Corvallis or Chailey	*qnrS1*			IncI1		ST-284	SMPA00000000	33	4984104
15	NAFTEC00039	SL_15_94	Raw chicken	Colombo	February 16, 2015	13	Agona	Agona	*fosA7*						SMPB00000000	32	4838005
16	NAFTEC00040	SL_16_97	Raw chicken	Colombo	February 16, 2015	1541	Corvalis	Corvallis or Chailey	*qnrS1*			IncI1		ST-284	SMPC00000000	36	4984004
17	NAFTEC00041	SL_17_98	Raw chicken	Colombo	February 16, 2015	1541	Corvalis	Corvallis or Chailey	*qnrS1*			IncI1		ST-284	SMPD00000000	34	4984047
18	NAFTEC00042	SL_18_102	Raw chicken	Colombo	February 16, 2015	13	Agona	Agona	*fosA7*			IncI1		ST-284	SMPE00000000	35	4978641
19	NAFTEC00043	SL_19_103	Raw chicken	Colombo	February 23, 2015	1541	Corvalis	Corvallis or Chailey	aph(3″)-Ib, aph(6)-Id, tet(A), qnrS1, sul2						SMPF00000000	33	4902599
20	NAFTEC00044	SL_20_107	Raw chicken	Colombo	February 23, 2015	1541	Corvalis	Corvallis or Chailey	*qnrS1*			IncI1		ST-284	SMPG00000000	35	4984004
21	NAFTEC00045	SL_21_109	Raw chicken	Colombo	February 23, 2015	13	Agona	Agona	*fosA7*						SMPH00000000	30	4838095
22	NAFTEC00046	SL_22_111	Raw chicken	Colombo	February 23, 2015	31	Newport	Newport							SMPI00000000	21	4903438
23	NAFTEC00047	SL_23_112	Raw chicken	Colombo	February 23, 2015	13	Agona	Agona	*fosA7*						SMPJ00000000	30	4836867
24	NAFTEC00048	SL_24_113	Raw chicken	Colombo	February 23, 2015	31	Newport	Newport							SMPK00000000	20	4660707
25	NAFTEC00049	SL_25_114	Raw chicken	Colombo	February 23, 2015	13	Agona	Agona	*fosA7*			IncI1		ST-284	SMPL00000000	34	4978594
26	NAFTEC00050	SL_26_115	Raw chicken	Colombo	February 23, 2015	1541	Corvalis	Corvallis or Chailey	*qnrS1*			IncI1		ST-284	SMPM00000000	35	4984783
27	NAFTEC00051	SL_27_116	Raw chicken	Colombo	February 23, 2015	31	Newport	Newport							SMPN00000000	20	4660888
28	NAFTEC00052	SL_28_117	Raw chicken	Colombo	February 23, 2015	314	Kentucky	Kentucky	*aph(6)-Id, tet(A), blaTEM-1B, qnrS1, sul3, dfrA14*			IncX1			SMPO00000000	33	4708122
29	NAFTEC00053	SL_29_118	Raw chicken	Colombo	February 23, 2015	1541	Corvalis	Corvallis or Chailey	*qnrS1*						SMPP00000000	30	4863328
30	NAFTEC00054	SL_30_119	Raw chicken	Colombo	February 23, 2015	314	Kentucky	Kentucky	*aph(6)-Id, tet(A), blaTEM-1B, qnrS1, sul3, dfrA14*			IncX1			SMPQ00000000	33	4708076
31	NAFTEC00055	SL_31_120	Raw chicken	Colombo	February 23, 2015	31	Newport	Newport							SMPR00000000	20	4660719
32	NAFTEC00056	SL_32_126	Raw chicken	Colombo	March 2, 2015	314	Kentucky	Kentucky	*aph(6)-Id, tet(A), blaTEM-1B, qnrS1, sul3*			IncX1			SMPS00000000	33	4705177
33	NAFTEC00057	SL_33_127	Raw chicken	Colombo	March 2, 2015	13	Agona	Agona	*fosA7*						SMPT00000000	35	4838212
34	NAFTEC00058	SL_35_S91	Human (blood)	Colombo	July 11, 2016	1541	Corvalis	Corvallis or Chailey	*qnrS1*						SMPU00000000	34	4855976
35	NAFTEC00059	SL_36_S106	Human (stool)	Jaffna	October 3, 2016	11	Enteritidis	Enteritidis				IncFIB(S)			SMPV00000000	24	4729498
36	NAFTEC00060	SL_37_S111	Human (stool)	Colombo	October 3, 2016	2063	Chester	Chester				IncFII(S)			SMPW00000000	26	4628679
37	NAFTEC00061	SL_38_S112	Human (blood)	Colombo	February 25, 2016	365	Welterveden	Weltevreden				IncFII(S)			SMPX00000000	63	4928542
38	NAFTEC00062	SL_39_S118	Human (stool)	Jaffna	October 3, 2016	1541	Corvalis	Corvallis or Chailey	*qnrS1*						SMPY00000000	32	4855244
39	NAFTEC00063	SL_40_S180	Human (blood)	Jaffna	October 13, 2016	11	Enteritidis	Enteritidis				IncFIB(S), IncFII(S)			SMPZ00000000	25	4705099
40	NAFTEC00064	SL_41_S215	Human (blood)	Jaffna	October 5, 2016	11	Enteritidis	Enteritidis				IncFIB(S), IncFII(S)			SMQA00000000	26	4705085
41	NAFTEC00065	SL_42_S216	Human (blood)	Jaffna	October 5, 2016	3771	Welterveden	Weltevreden				IncFII(S)			SMQB00000000	62	4916302
42	NAFTEC00066	SL_43_S218	Human (blood)	Colombo	October 15, 2016	11	Enteritidis	Enteritidis		*D87G*		IncFIB(S), IncFII(S)			SMQC00000000	24	4707867
43	NAFTEC00067	SL_44_S232	Human (stool)	Colombo	October 13, 2016	11	Enteritidis	Enteritidis				IncFIB(S), IncFII(S)			SMQD00000000	27	4738730
45	NAFTEC00069	SL_46_S250	Human (blood)	Colombo	October 13, 2016	11	Enteritidis	Enteritidis		*D87Y*		IncFIB(S), IncFII(S)			SMQF00000000	25	4646372
46	NAFTEC00070	SL_47_S271	Human (blood)	Galle	October 9, 2016	11	Enteritidis	Enteritidis		*D87Y*		IncFIB(S), IncFII(S)			SMQG00000000	23	4704679
47	NAFTEC00071	SL_48_S290	Human (stool)	Ragama	October 24, 2016	11	Enteritidis	Enteritidis				IncFIB(S), IncFII(S)			SMQH00000000	24	4729267
49	NAFTEC00073	SL_50_S294	Human (blood)	Colombo	October 24, 2016	43	Paratyphi B var java	Paratyphi B							SMQJ00000000	37	4753025
50	NAFTEC00074	SL_51_S295	Human (blood)	Colombo	October 24, 2016	11	Enteritidis	Enteritidis				IncFIB(S), IncFII(S)			SMQK00000000	25	4729886
51	NAFTEC00075	SL_52_S304	Human (stool)	Ragama	November 2, 2016	11	Enteritidis	Enteritidis				IncFIB(S), IncFII(S)			SMQL00000000	24	4729267
52	NAFTEC00076	SL_53_S307	Human (blood)	Colombo	November 7, 2016	287	Mountpleasant	Mountpleasant	*fosA7*						SMQM00000000	30	4645898
53	NAFTEC00077	SL_55_S309	Human (blood)	Colombo	November 10, 2016	43	Paratyphi B var java	Paratyphi B							SMQN00000000	38	4753099
54	NAFTEC00078	SL_56_S314	Human (blood)	Kandy	November 24, 2016	11	Enteritidis	Enteritidis		*D87G*		IncFIB(S), IncFII(S)	[F-:A16:B22]		SMQO00000000	24	4707889
55	NAFTEC00079	SL_57_S315	Human (blood)	Kandy	November 24, 2016	11	Enteritidis	Enteritidis		*D87G*		IncFIB(S), IncFII(S)	[F-:A16:B22]		SMQP00000000	27	4707714
56	NAFTEC00080	SL_58_S327	Human (stool)	Jaffna	November 29, 2016	3771	Welterveden	Weltevreden				IncFII(S)	[S1:A-:B-]		SMQQ00000000	68	4932856
57	NAFTEC00081	SL_59_S329	Human (blood)	Colombo	November 30, 2016	3771	Welterveden	Weltevreden				IncFII(S)	[F-:A16:B-]		SMQR00000000	68	4916026
58	NAFTEC00082	SL_61_S333	Human (blood)	Colombo	December 6, 2016	11	Enteritidis	Enteritidis				IncFIB(S), IncFII(S)	[F-:A16:B22]		SMQS00000000	26	4705042
59	NAFTEC00083	SL_62_S353	Human (joint fluid)	Colombo	December 17, 2016	1541	Corvalis	Corvallis or Chailey							SMQT00000000	31	4894143
60	NAFTEC00084	SL_63_S360	Human (blood)	Ragama	December 22, 2016	1602	Mbandaka	Mbandaka							SMQU00000000	36	4722738
61	NAFTEC00085	SL_64_D94	Human (stool)	Colombo	December 30, 2016	2063	Chester	Chester	*aph(6)-Id, blaTEM-1B, qnrS1, sul3, dfrA14*			IncX1			SMQV00000000	33	4586888
62	NAFTEC00086	SL_65_D912	Human (stool)	Awissawella	December 30, 2016	2063	Chester	Chester	*aph(6)-Id, blaTEM-1B, qnrS1, sul3, dfrA14*			IncX1			SMQW00000000	35	4587278
63	NAFTEC00087	SL_66_D001	Human (stool)	Colombo	January 5, 2017	29	Stanley	Stanley							SMQX00000000	24	4633473
64	NAFTEC00088	SL_67_S04	Human (stool)	Peradeniya	January 9, 2017	11	Enteritidis	Enteritidis			*E466D*	IncFIB(S), IncFII(S)	[F-:A16:B22]		SMQY00000000	26	4706648
66	NAFTEC00090	SL_69_S11	Human (stool)	Jaffna	January 9, 2017	36	Typhimurium	Typhimurium	*blaCTX-M-15*			IncI1		ST-31	SMRA00000000	36	4723314
67	NAFTEC00091	SL_70_W02	Human (stool)	Galle	February 13, 2017	−5309^h^	Vancouver	Vancouver							SMRB00000000	33	4645994
68	NAFTEC00092	SL_71_W03	Human (stool)	Galle	February 13, 2017	11	Enteritidis	Enteritidis				IncFIB(S), IncFII(S)	[F-:A16:B22]		SMRC00000000	25	4729763
69	NAFTEC00093	SL_72_S24	Human (blood)	Badulla	February 13, 2017	11	Enteritidis	Enteritidis		*D87G*		IncFIB(S), IncFII(S)	[S1:A-:B22]		SMRD00000000	24	4707889
70	NAFTEC00094	SL_73_S41	Human (blood)	Galle	February 25, 2017	11	Enteritidis	Enteritidis				IncFIB(S), IncFII(S)	[F-:A16:B22]		SMRE00000000	25	4705684
71	NAFTEC00095	SL_74_D64	Human (stool)	Kandy	February 13, 2017	43	Paratyphi B var java	Paratyphi B							SMRF00000000	38	4753322
72	NAFTEC00096	SL_75_D66	Human (stool)	Colombo	February 13, 2017	43	Paratyphi B var java	Paratyphi B							SMRG00000000	37	4753109
75	NAFTEC00099	SL_78_S58	Human (blood)	Colombo	February 13, 2017	11	Enteritidis	Enteritidis							SMRJ00000000	21	4587841
76	NAFTEC00100	SL_79_S78	Human (blood)	Kandy	February 27, 2017	11	Enteritidis	Enteritidis				IncFIB(S), IncFII(S)	[F-:A16:B22]		SMRK00000000	23	4705408
77	NAFTEC00101	SL_80_S79	Human (blood)	Kandy	February 27, 2017	11	Enteritidis	Enteritidis				IncFIB(S), IncFII(S)	[F-:A16:B22]		SMRL00000000	25	4789497
79	NAFTEC00103	SL_82_D95	Human (stool)	Awissawella	February 27, 2017	11	Enteritidis	Enteritidis				IncFIB(S), IncFII(S)	[F-:A16:B22]		SMRN00000000	26	4707602

^a^ Using MLST v2.0.

^b^ Performed serological identification according to Kauffman–White scheme.

^c^ Using SeqSero v1.0.

^d^ Using ResFinder v2.3 (minimum percentage identity of 90% and minimum length of 60%).

^e^ Using PlasmidFinder 1.3 (minimum percentage identity of 95% and minimum length of 60%).

^f^ Using pMLST v2.0.

^g^ Using Quast v4.6.3.

^h^ Isolate with new ST being assigned by EnteroBase.

MLST, Multilocus sequence typing; ST, sequence type.

Genomic analyses showed that *Salmonella enterica* serovar Agona of ST13 (*n* = 16) and *Salmonella enterica* serovar Enteritidis of ST11 (*n* = 21) were the most prevalent serovars that were observed among chicken meat and human isolates, respectively. *Salmonella enterica* serovar Corvallis ST1541 is the only serovar that was overlapping between human and chicken meat in this study. There was 100% concordance between conventional serotyping by Kauffman–White scheme and genotypic serotype prediction by SeqSero (Zhang *et al.*, 2015). Discrepancy was observed for four human isolates; they were serotyped to be Paratyphi B var java (henceforth Java) but were predicted to be Paratyphi B. They have identical serological formula and Java is considered a variant of Paratyphi B that can ferment d-tartrate, whereas Paratyphi B cannot due to a single nucleotide change in the start codon of the STM3356 gene (Malorny *et al.*, 2003). Hence, when the draft genome of these four isolates were blasted against the STM3356 gene of Java strain NCTC5706 (GenBank accession number: LT571437.1), the start codon was ATG (data not shown). In addition, these isolates were phenotypically tested to be positive for d-tartrate fermentation (data not shown). Altogether, this indicates the isolates are able to ferment d-tartrate and they are indeed Paratyphi B var. Java, which tallies with the serotyping result. Hence, this suggests that additional genetic loci or alleles should be taken into consideration for prediction of a certain serotype from sequence data. More than two-thirds (50/73) of the isolates contained plasmid replicons. The commonly seen plasmid replicons were IncFII(S) and IncFIB(S), of sequence type [F-:A16:B22] and were found in 16 *Salmonella* Enteritidis strains. Among the chicken meat isolates, 87.9% (29/33) of them had at least one resistance gene and multidrug resistance (MDR; defined as resistance to three or more classes of antibiotics) was observed in 15.2% of them (5/33), including two *Salmonella enterica* serovar Kentucky ST314 strains, which harbored six different classes of antimicrobial resistance (AMR) determinants. In contrast, among the human isolates, 17.5% (7/40) of them had at least one resistance gene and only two isolates (5%, 2/40) were found to be MDR, which were both *Salmonella enterica* serovar Chester ST2063 strain that contained five AMR determinants, belonging to five different antibiotic classes. It is worth mentioning that out of 73 isolates, only one human isolate contained extended-spectrum beta-lactamase (ESBL) gene, bla_CTX-M-15_. As expected, when we performed the double-disk synergy test (Guo *et al.*, 2019), the strain was tested to be positive for ESBL production. Among all the identified AMR genes, the most frequent resistance genotype was *fosA7* and was found in all 16 *Salmonella* Agona (ST13) strains from different chicken meat samples. We did not test the phenotypic resistance of these isolates to fosfomycin, and hence we do not know if *fosA7* gene confers phenotypic resistance to fosfomycin. When a whole-genome single nucleotide polymorphism (SNP) analysis with CFSAN SNP Pipeline (Davis *et al.*, 2015) that was installed on GalaxyTrakr (https://www.galaxytrakr.org) (Afgan *et al.*, 2018) was performed on the 16 *Salmonella* Agona isolates, the minimum and maximum SNP differences were 0 and 36, respectively (data not shown). Upon construction of the best-scoring maximum likelihood (ML) SNP tree with randomized axelerated ML (RAxML) using a GTRGAMMA model of evolution and default parameters (Stamatakis, 2014), it appears that some isolates are phylogenetically related due to 0 SNP difference, but they may not be epidemiologically related due to lack of information on sampling source.

The investigation has identified the *S*. *enterica* serovars that were dominating in chicken meat and human, and showed the genomics differences among the chicken meat and human strains. Since it is a retrospective study, it is limited by the absence of adequate (≥50) and regular sampling at indicated cities/locations for a longer period of time (≥1 year), within the same time period for both chicken meat and human samples. Hence, it is not possible to draw any conclusion about the correlation between the clinical isolates and the chicken reservoir. Nevertheless, the generated data do provide very rough details about *Salmonella* serotypes and resistance traits in chicken meat and human in studied cites, and contribute to the design of sampling framework for prospective *Salmonella* and AMR surveillance.

## References

[B1] Afgan E, Baker D, Batut B, *et al.* The Galaxy platform for accessible, reproducible and collaborative biomedical analyses: 2018 update. Nucleic Acids Res 2018;46:W537–W544 29790989 10.1093/nar/gky379 PMC6030816

[B2] Alikhan NF, Zhou Z, Sergeant MJ, Achtman M A genomic overview of the population structure of Salmonella. PLoS Genet 2018;14:e1007261 29621240 10.1371/journal.pgen.1007261 PMC5886390

[B3] Carattoli A, Zankari E, Garcia-Fernandez A, *et al.* In silico detection and typing of plasmids using PlasmidFinder and plasmid multilocus sequence typing. Antimicro Agents Chemother 2014;58:3895–3903 10.1128/AAC.02412-14 PMC4068535 24777092

[B4] Crump JA, Sjolund-Karlsson M, Gordon MA, Parry CM Epidemiology, clinical presentation, laboratory diagnosis, antimicrobial resistance, and antimicrobial management of invasive *Salmonella* infections. Clin Microbiol Rev 2015;28:901–937 26180063 10.1128/CMR.00002-15 PMC4503790

[B5] Davis S, Pettengill JB, Luo Y, *et al.* CFSAN SNP Pipeline: An automated method for constructing SNP matrices from next-generation sequence data. PeerJ Comput Sci 2015;1:e20

[B6] Feasey NA, Dougan G, Kingsley RA, Heyderman RS, Gordon MA Invasive non-typhoidal salmonella disease: An emerging and neglected tropical disease in Africa. Lancet 2012;379:2489–2499 22587967 10.1016/S0140-6736(11)61752-2 PMC3402672

[B7] Guo S, Tay MYF, Aung KT, *et al.* Phenotypic and genotypic characterization of antimicrobial resistant Escherichia coli isolated from ready-to-eat food in Singapore using disk diffusion, broth microdilution and whole genome sequencing methods. Food Control 2019;99:89–97

[B8] Gurevich A, Saveliev V, Vyahhi N, Tesler G QUAST: Quality assessment tool for genome assemblies. Bioinformatics 2013;29:1072–1075 23422339 10.1093/bioinformatics/btt086 PMC3624806

[B9] Larsen MV, Cosentino S, Rasmussen S, *et al.* Multilocus sequence typing of total-genome-sequenced bacteria. J Clin Microbiol 2012;50:1355–1361 22238442 10.1128/JCM.06094-11 PMC3318499

[B10] Malorny B, Bunge C, Helmuth R Discrimination of d-tartrate-fermenting and -nonfermenting *Salmonella enterica* subsp. enterica isolates by genotypic and phenotypic methods. J Clin Microbiol 2003;41:4292–4297 12958259 10.1128/JCM.41.9.4292-4297.2003 PMC193836

[B11] Ministry of Health. Weekly Epidemiological Report. Volume 2018. Sri Lanka: Epidemiology Unit, Ministry of Health, 2018

[B12] Rowe B, Ward LR, Threlfall EJ Multidrug-resistant Salmonella typhi: A worldwide epidemic. Clin Infect Dis 1997;24 Suppl 1:S106–S109 8994789 10.1093/clinids/24.supplement_1.s106

[B13] Stamatakis A RAxML version 8: A tool for phylogenetic analysis and post-analysis of large phylogenies. Bioinformatics 2014;30:1312–1313 24451623 10.1093/bioinformatics/btu033 PMC3998144

[B14] Tay MYF, Adzitey F, Sultan SA, Tati JM, Seow KLG, and Schlundt J Whole-genome sequencing of nontyphoidal *Salmonella enterica* isolates obtained from various meat types in Ghana. Microbiol Res Anncmnts 2019;8 10.1128/MRA.00033-19 PMC6460018 30975795

[B15] Zankari E, Hasman H, Cosentino S, *et al.* Identification of acquired antimicrobial resistance genes. J Antimicrob Chemother 2012;67:2640–2644 22782487 10.1093/jac/dks261 PMC3468078

[B16] Zhang S, Yin Y, Jones MB, *et al.* Salmonella serotype determination utilizing high-throughput genome sequencing data. J Clin Microbiol 2015;53:1685–1692 25762776 10.1128/JCM.00323-15 PMC4400759

